# Correction: Hardy et al. Genome Sequence and Characterization of Five Bacteriophages Infecting *Streptomyces coelicolor* and *Streptomyces venezuelae*: Alderaan, Coruscant, Dagobah, Endor1 and Endor2. *Viruses* 2020, *12*, 1065

**DOI:** 10.3390/v13081616

**Published:** 2021-08-16

**Authors:** Aël Hardy, Vikas Sharma, Larissa Kever, Julia Frunzke

**Affiliations:** Institute of Bio- and Geosciences, IBG-1: Biotechnology, Forschungszentrum Jülich, 52425 Jülich, Germany; a.hardy@fz-juelich.de (A.H.); v.sharma@fz-juelich.de (V.S.); l.kever@fz-juelich.de (L.K.)

The authors wish to make the following corrections to this paper [1]:

Replacement of the picture showing the plaque morphology of the phage Coruscant (Figure 1A), as well as of the growth infection curves (Figure 2), after we noticed a contamination in our phage stocks. The corrected Figures are listed below. 

Additionally, we would like to indicate that we made the following changes in the text of the manuscript:

- Section 2.4

Section 2.4 was as follows:

“Infection in shake flasks (*S. venezuelae* phages): 70 mL GYM medium were inoculated with 10^5^ spores and incubated at 30 °C for 6–8 h to allow spore germination. Phages were then added at the corresponding multiplicity of infection (MOI). OD_450_ was measured over time to assess bacterial growth. In parallel, the filtered supernatants of the cultures were collected at the same time points. 3 µL of these supernatants were spotted on a *Streptomyces venezuelae* lawn (inoculated to an OD_450_ = 0.4) at the end of the experiment to estimate the phage titer. 

Infection in microtiter plates (*S. coelicolor* phages): Growth experiments were performed in the BioLector^®^ microcultivation system of m2p-labs (Aachen, Germany). Cultivation was performed as biological triplicate sin 48-well FlowerPlates (m2plabs) at 30 °C and a shaking frequency of 1200 rpm [21]. Backscatter was measured by scattered light with an excitation wavelength of 620 nm (filter module: λ_Ex_/λ_Em_: 620 nm/620 nm, gain: 25) every 15 min. Each well contained 1 mL YEME medium and was inoculated with 10^6^ spores of *S. coelicolor* M145. Phages were added after 7 h, and sampling was performed at the indicated time points. Subsequently, 2 µL of the supernatants were spotted on a lawn of *S. coelicolor* propagated on a double overlay of GYM agar inoculated at an initial OD_450_ = 0.4.”

We replaced it with the following text:

“Growth experiments were performed in the BioLector^®^ microcultivation system of m2p-labs (Aachen, Germany). Cultivation was performed as biological triplicates in 48-well FlowerPlates (m2plabs) at 30 °C and a shaking frequency of 1200 rpm [21]. Backscatter was measured by scattered light with an excitation wavelength of 620 nm (filter module: λ_Ex_/λ_Em_: 620 nm/620 nm, gain: 25) every 15 min. Each well contained 1 mL YEME or GYM medium and was inoculated using an overnight culture of *S. coelicolor* or *S. venezuelae*, respectively, to an initial OD_450_ of 0.1. Phages were directly added to an initial titer of 10^5^, 10^6^, or 10^7^ PFU/mL, and sampling was performed at the indicated time points. Subsequently, 2 µL of the supernatants were spotted on a lawn of *S. coelicolor* or *S. venezuelae* propagated on a double overlay of GYM agar inoculated at an initial OD_450_ = 0.5.”

- Section 2.6

We replaced the concentration of proteinase K indicated as 50 mM with 50 µg/mL.

We corrected the volume of water used to resuspend DNA pellets from 3 to 30 µL.

- Section 3.1

We replaced the following sentence:

“The phages Alderaan and Coruscant were isolated using *Streptomyces venezuelae* ATCC 10712 and formed small, transparent and round plaques of approximately 2 mm of diameter (Figure 1A).”

With the one below:

“The phages Alderaan and Coruscant were isolated using *Streptomyces venezuelae* ATCC 10712. Alderaan formed small, transparent, and round plaques of approximately 2 mm of diameter, while the plaques formed Coruscant were very small (<1 mm) and were fully visible only after 2 days of incubation (Figure 1A).”

- Section 3.2

The description of the phage infection curves was as follows: 

“Phage infection in liquid cultures was performed to assess infection dynamics. Due to the complex developmental cycle of *Streptomyces*, standard one-step growth curves could not be performed. We instead inoculated liquid cultures with spores of *Streptomyces* and let them germinate for approximately 7 h before adding the phages to a multiplicity-of-infection (MOI) from 0.1 to 10. For the *S. venezuelae* phages, infection was performed in flasks and OD_450_ was used to estimate cell density. In contrast, *S. coelicolor* was cultivated in microtiter plates, and cell growth was monitored using continuous backscatter measurements. In both cases, phage titer was measured over time to estimate the production of phage progeny.

Infection of *S. venezuelae* with Alderaan and Coruscant showed moderate lysis for MOI 1, and distinct OD drops for the MOI 10, which was reduced to almost zero after 24 h of infection (Figure 2A). Phage titers showed a significant increase after 16 h of infection and were markedly higher for MOI 10 than MOI 1. 

As for the *S. coelicolor* phages (Figure 2B), infection with Dagobah caused a mild growth delay, visible especially for the highest MOI (MOI 1). In parallel, the phage titers grew moderately (10^2^-fold increase between 0 and 48 h) or strongly (10^5^-fold increase between 0 and 48 h) for initially low (MOI 0.05) or high (MOI 1) MOIs, respectively. In contrast, infection with Endor1 had a profound effect on bacterial growth, as the highest MOIs (MOI 0.1 and 1) effectively suppressed growth. The phage titers showed concordant behavior, with a strong increase from 16 h and a titer plateauing at a high level for MOI 0.1. Endor2 showed an intermediate effect: the growth curves were significantly shifted, proportionally to the initial MOIs. At low MOIs, the evolution of Endor1/2 titer was bell-shaped, with an initial increase until 40 h followed by a decline down to a virtually null titer at the end of the experiment.

Furthermore, the backscatter started to decrease in the uninfected wells starting from 50 h, coinciding with the start of the production of blue-pigmented actinorhodin. A similar drop was also observed in the samples infected with Dagobah, Endor1 and the lowest MOI of Endor2.

Altogether, infection curves revealed that all five phages can successfully propagate in liquid cultures at the expense of their host. Surprisingly, the titers of phages Endor1 and Endor2 dropped after an initial increase, which needs further investigation.”

It has been replaced by the description below:

“Phage infection in liquid cultures was performed to assess infection dynamics. Due to the complex developmental cycle of *Streptomyces*, standard one-step growth curves could not be performed. Instead, we cultivated *S. coelicolor* and *S. venezuelae* in microtiter plates in presence of phage challenge, and cell growth was monitored over a 24 h time period using continuous backscatter measurements. In both cases, phage titer was measured over time to estimate the production of phage progeny.

Infection of *S. venezuelae* with Alderaan showed a marked culture collapse at the highest initial phage load (10^7^ PFU/mL), and a plateauing of cell biomass at a significantly reduced level for the intermediate phage challenge (10^6^ PFU/mL). In contrast, addition of Coruscant causes only a mild but initial titer-dependent growth delay of the cultures (Figure 2A). For both phages, phage titers peaked at 6 h at the higher initial phage titer (10^7^ PFU/mL), and at the intermediate phage challenge, phage amplification was delayed or very weak for Alderaan and Coruscant, respectively.

As for the *S. coelicolor* phages (Figure 2B), infection with Dagobah caused a mild growth delay, visible especially when 10^7^ PFU/mL was initially added. In parallel, the phage titers either declined over time or grew moderately (10-fold increase between 0 and 8 h) for initially intermediate (10^6^ PFU/mL) or high (10^7^ PFU/mL) phage challenge, respectively. Infection with Endor1 and Endor2 showed a similar behavior and caused a stronger growth delay than Dagobah, even for the intermediate initial phage burden (10^6^ PFU/mL). The phage titers showed concordant behavior, with a strong increase in titers for both Endor1 and Endor2 until 10 h, followed by a marked decline up to 24 h.

Altogether, infection curves revealed that all five phages can successfully propagate in liquid cultures at the expense of their host. Surprisingly, the titers of all phages dropped after an initial increase, which needs further investigation.”

- Section 3.3

We corrected the genome size of phage Alderaan from 34 to 39 kb (Table 2 and corresponding text).

The authors would like to apologize for any inconvenience caused by these changes to the readers.

## Figures and Tables

**Figure 1:**
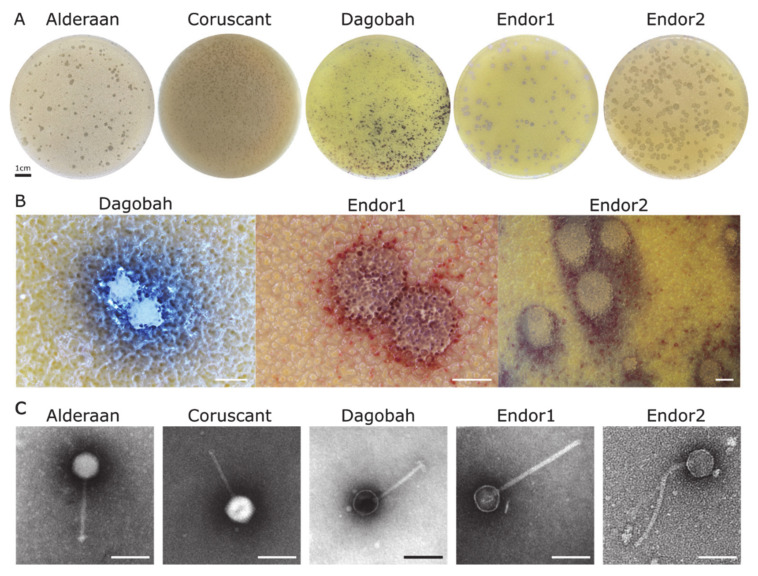
Morphology observation of five novel *Streptomyces* phages. (**A**) Plaque morphologies of the five phages. Double agar overlays were performed to infect *S. venezuelae* ATCC 10712 with the phages Alderaan and Coruscant, and *S. coelicolor* M600 with the phages Dagobah, Endor1, and Endor2. Plates were incubated overnight at 30 °C and another day (3 days in the case of Dagobah) at room temperature to reach full maturity of the bacterial lawn. (**B**) Close-ups of phage plaques imaged using a stereomicroscope Nikon SMZ18. *S. coelicolor* M145 was infected by phages using GYM double agar overlays. The plates were incubated at 30 °C overnight and then kept at room temperature for two (Endor1 and Endor2) or three days (Dagobah). Scale bar: 1 mm. (**C**) Transmission electron microscopy (TEM) of phage isolates. The phage virions were stained with uranyl acetate. Scale bar: 150 nm.

**Figure 2:**
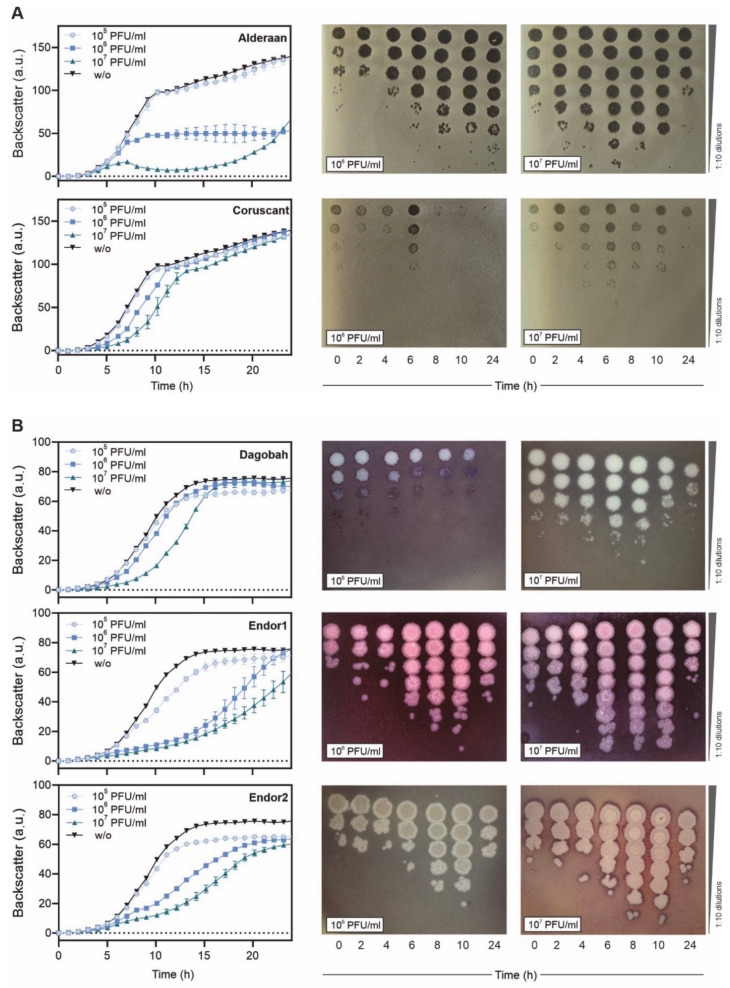
Infection curves of the five phages infecting *S. venezuelae* (**A**) and *S. coelicolor* (**B**). *S. venezuelae* or *S. coelicolor* were inoculated to GYM or YEME medium, respectively, and grown in microtiter plates, to which phages were added at the indicated initial phage titers. Backscatter was measured over time (left panels), in parallel to phage titers (right panels).
